# Socioeconomic factors predictive of access delays in oncology

**DOI:** 10.1186/s12889-025-23398-5

**Published:** 2025-06-10

**Authors:** Allen M. Chen

**Affiliations:** Department of Radiation Oncology, Chao Family Comprehensive Cancer Center, Orange, University of California, 101 The City Drive, Building 23, Irvine, CA 92868 USA

**Keywords:** Health services, Disparities, Access, Social determinants, Inclusion

## Abstract

**Purpose:**

To identify demographic and socioeconomic factors predictive of access delays in patients referred for radiation oncology services.

**Methods and materials:**

A prospective data registry of consecutive patients referred for initial consultation from October 2018 to April 2022 was reviewed. To evaluate access, the number of business days from referral to consultation was calculated. Demographic characteristics recorded included age, gender, race, language preference, and insurance status. Zip code data linked to a patient’s residential address was used to classify socioeconomic status (SES) based on publicly available data on median household income. Descriptive statistics were presented to identify factors predictive of delays in the time from referral to consultation.

**Results:**

A total of 9,241 consecutive patients were referred and logged into the database during the 4-year period, of which 5,321 were scheduled, registered, and seen in the outpatient setting. Delays in access were associated with low SES and Black race (*p* < 0.05, for both). Three hundred and seventeen of the 1,203 patients (26%) in the lowest SES quartile had their appointments scheduled greater than 21 days from the time of referral compared to 482 of the 4,118 patients (12%) in the non-lowest quartile SES group (*p* < 0.001). Black patients were significantly less likely to have their appointments within 5 days compared to non-Black patients (17% versus 24%, *p* = 0.01). On multivariate analysis, the only variable independently associated with higher odds of appointment delays was SES (low-SES quartile versus non-low-SES quartile (OR = 3.98, 95% CI [2.01–7.92], *p* < 0.001).

**Conclusions:**

SES factors related to geographical zip code predict for access delays in radiation oncology care. Targeted interventions are urgently warranted for low SES groups residing in underserved communities.

## Introduction

Access to healthcare— defined by the National Academy of Medicine as"the timely use of personal health services to achieve the best health outcome” is a fundamental cornerstone of any society [1]. For patients with a new diagnosis of cancer, the importance of obtaining a provider appointment as expeditiously as possible has been well-established by studies associating improved clinical results with earlier initiation of treatment [2]. One systematic review of 34 studies across 7 cancer types showed that every month delay in starting treatment was associated with an approximate 10% increase in mortality [3]. In this meta-analysis, some of the most pronounced detrimental effects were seen in patients referred for radiation therapy compared to those referred for surgery and/or chemotherapy. However, it is uncertain which patients might be most susceptible to treatment delays as a result of difficulties in appointment scheduling and there is need to identify risk factors for access impairment. Although investigations have attempted to correlate various patient-related measures with metrics related to access in healthcare, these have generally focused on primary care or non-oncology specialties [4–6]. Additionally, the findings have been relatively inconsistent with age, insurance status, ethnicity, and location commonly cited as possible influential factors. The purpose of this study was to therefore perform a population-based analysis of demographic and socioeconomic factors which might be predictive for delays in care among patients referred for radiation therapy after a diagnosis of cancer.

## Methods

### Study design

From October 2018 to April 2022, patients referred to a tertiary-based Department of Radiation Oncology affiliated with a National Cancer Institute (NCI)-designated Comprehensive Cancer Center were inputted into a commercially available, enterprise-based electronic medical record system (Epic Systems, Inc. Verona, WI) for assignment to physicians for initial consultation. This information was used to prospectively populate a customized registry using data dictionaries and included fields allowing for the collection of patient-specific demographic and disease characteristics. Only patients with a known diagnosis of cancer at the time of referral were included. Population-based data based on demographic information such as sex, ethnicity, and race were categorized using standard nomenclature in accordance with that determined by the United States Census Bureau [7]. Given that ethnicity is a characterization based on shared culture expression, the decision was made to use race for purposes of categorization. In this analysis, race was based on self-identification at the time of intake and officially recognized with 5 racial categories (White, Black, Asian, Latino, Native Hawaiian/Pacific Islander). Patients who self-identified with more than 1 race or who preferred not to disclose this information were grouped into the “other” category. Zip code data linked to a patient’s residential address was used to determine socioeconomic status (SES) based on publicly available data on median household income extracted from the California Open Data Portal [8]. This dataset, published using information from California Franchise Tax Board, contained data from California resident tax returns filed with California adjusted gross income and self-assessed tax listed by zip code. This dataset contained data for taxable years 1992 to the most recent tax year available. Based on this dataset, SES was subsequently categorized into 4 designated quartiles (based on the zip codes extracted from the study cohort) which correlated with income level (low, medium–low, medium–high, and high) as determined by patient zip code. Insurance status was classified into public versus private. Those without insurance and/or who were self-insured were specifically excluded. Patients were deemed non-English speaking if requiring translator services for direct communication with providers. This analysis focused specifically on barriers related to the scheduling of new consultation appointments and did not include follow-up encounters.

### Study cohort

**Fig. 1:**
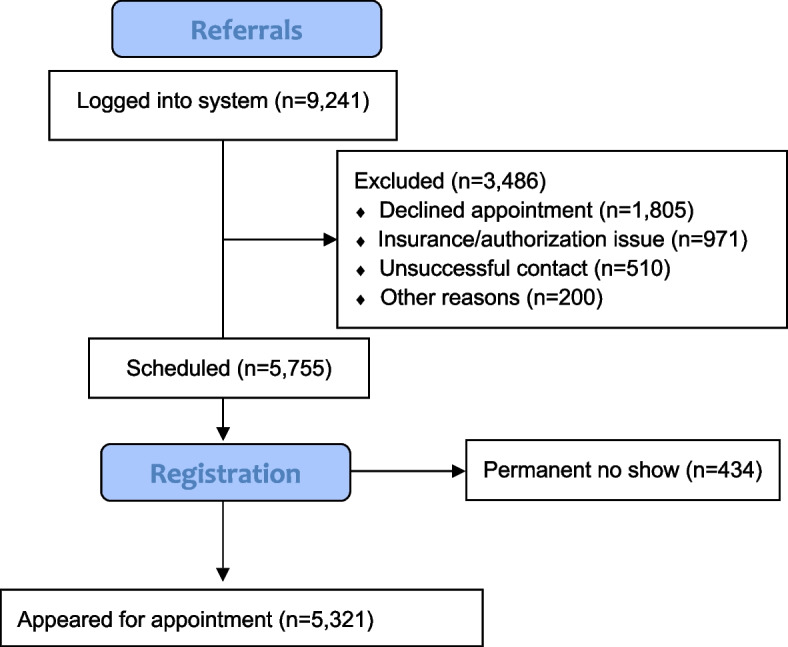
Flow diagram to illustrate determination of the primary study population

A total of 9,241 consecutive patients were formally referred and logged into the database during the 4-year period, of which 5,755 were successfully scheduled and registered. The remaining patients were not seen in consultation and either declined to be treated and/or seen in consultation, or alternatively, received radiation therapy elsewhere. Among the 5,755 patients scheduled for initial consultation, a total of 523 patients (9%) failed to show for their appointments of which 89 were successfully re-scheduled and subsequently attended their appointment. The 5,321 patients who actually appeared for their consultation appointments formed the primary population of this analysis. Figure 1 depicts a flow diagram illustrating how the subject population was derived.

### Endpoints and statistical analysis

Descriptive frequencies of demographic and SES characteristics associated with patient delays, as determined by measuring the time (in business days) from referral to consultation, were assessed. The proportion of patients who were seen for their consultation appointment within 5, 7, 14, and 21 days was analyzed, with the numerical thresholds selected based on the work of others [9, 10]. Categorical variables were compared using chi-squared statistics for frequency and proportions. Continuous variables were presented as means and compared using t-tests. The significance level was set at 0.05 for all analyses. To investigate correlates of appointment non-adherence, multivariable logistic regression analyses were performed assessing for age, sex, insurance, and SES. Low-income designation included patients from zip codes categorized as low-income or medium–low status. Distance from residence to the treatment facility was calculated in miles. The selection of adjusting factors for multivariable analysis was made based on univariate analysis. Given the potential class imbalance in events across variables, standardized oversampling techniques were employed to verify the findings of the logistic regression. All statistical analysis was performed on SAS, version 9.4 (SAS Institute, Cary, NC).

## Results

Primary disease sites were breast (N = 1,176), prostate (N = 988), head/neck/skin (N = 739), lung (N = 567), gastrointestinal (N = 557), gynecologic (N = 399), central nervous system (N = 275), hematologic (N = 99), other/miscellaneous (N = 521). The median age of the patients scheduled for consultation was 55 years (range, 17 to 101). Gender was 2,790 male (52%); 2,531 female (48%); Race was 3,001 White (56%); 944 Latino (18%); 942 Asian (18%); 389 Black (7%); and 45 Native American/Pacific Islander/Other (1%). Eight hundred and forty-nine patients (16%) were non-English speaking. A total of 112 zip codes were used to classify patients into 4 categories of 28 zip codes each to create distinct quartiles for the purpose of analyzing SES based on residential address. All 112 zip codes were from California. Using the census data, the median household incomes associated with each zip code ranged from $27,683 to $475,757 (mean, $78,570). The four created quartiles were disturbed as follows: low, below $49,999; medium–low, $50,000 to $78,570; medium–high, $78,571 to $101,999; high, $102,000 and above. Insurance status was categorized as follows: 2407 private (commercial) insurance; 2914 public insurance.

**Fig. 2:**
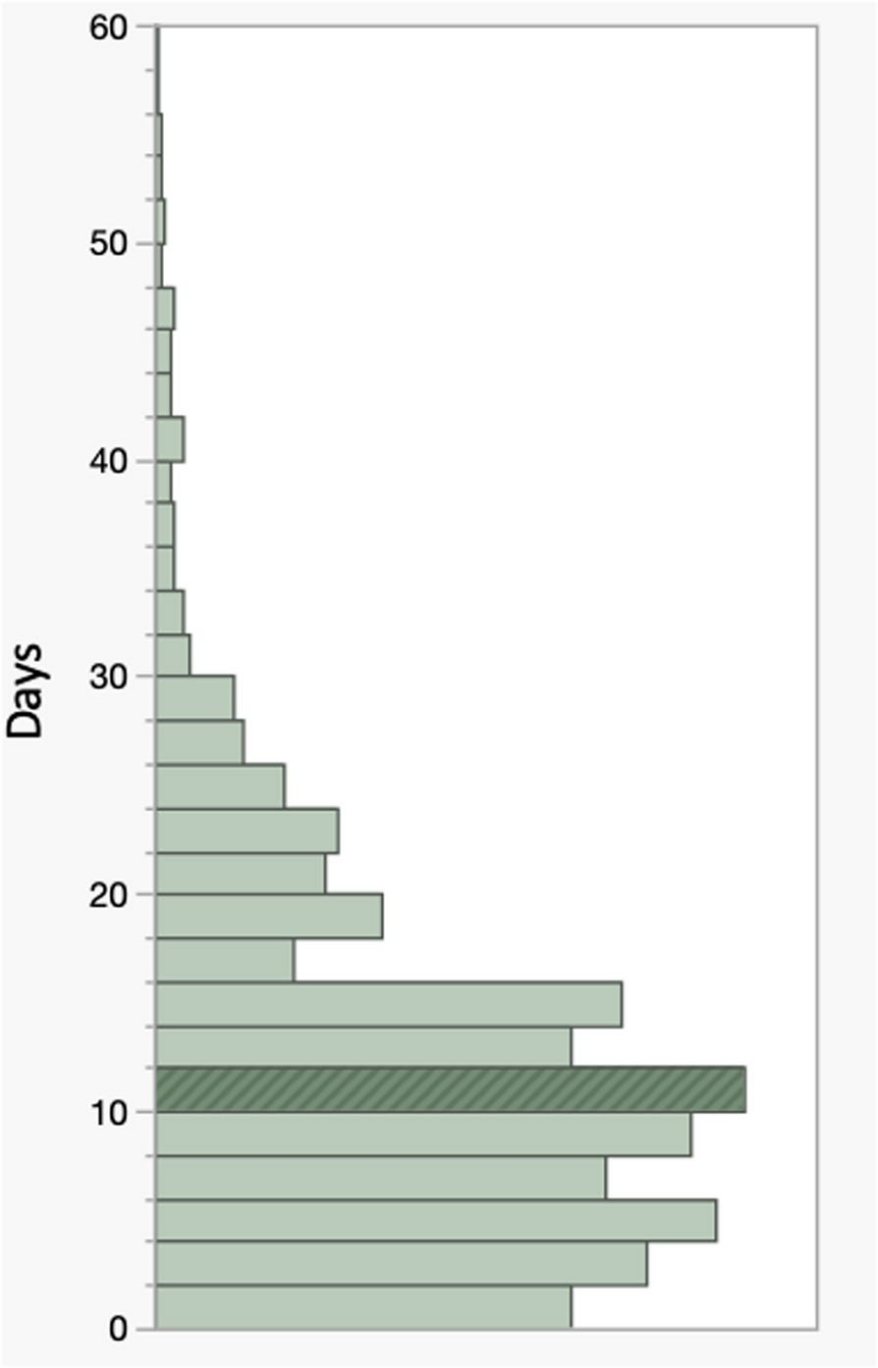
Histogram illustrating the distribution of time interval from referral to consultation

Among the 5,321 patients who successfully completed their visit, the median time from referral to the date of appointment was 10 days (range, 0 to 149 days). The proportion of patients who were seen for their consultation appointment within 5, 7, 14, and 21 days was 23% (n = 1223), 37% (n = 1968), 73% (n = 3884), and 85% (n = 4522), respectively. Figure 2 illustrates the distribution for the entire population with respect to time from referral to consultation (business days) in histogram format.

As illustrated in Table 1, the variables that predicted for excessive delays in patient appointments were SES categorization and race defined by income (*p* < 0.05, for both). Three hundred and seventeen of the 1,203 patients (26%) in the lowest SES quartile completed their appointments greater than 21 days from the time of referral compared to 482 of the 4,118 patients (12%) in the non-lowest quartile SES group (*p* < 0.001). Conversely, 401 of the 1,379 patients (29%) in the highest quartile SES quartile were able to be seen for consultation within 5 days compared to only 130 of the 1,203 patients (11%) in the lowest SES quartile (*p* < 0.001). The proportion of White, Latino, Asian, and Black patients who had their appointment more than 21 days from the time of referral was 16%, 13%, 14%, and 16%, respectively (*p* = 0.45). However, Black patients were significantly less likely to have their appointments within 5 days compared to non-Black patients (17% versus 24%, *p* = 0.01). None of the other factors analyzed including language preference, age, gender, or insurance status predicted for access delays (p > 0,05, for all). The distance from residence to the treatment facility, as measured in miles as a continuous variable, did not predict for delays in access (*p* = 0.35).

**Table 1:** Potential demographic and socioeconomic factors predictive of access

	< 5 days (*n* = 1223)	5–7 days (*n* = 745)	8–14 days (*n* = 1916)	15–21 days (*n* = 638)	> 21 Days (*n* = 799)
Race (*p* = 0.01)					
White	709 (24)	476 (16)	930 (31)	404 (13)	482 (16)
Latino	214 (23)	109 (12)	400 (42)	96 (10)	125 (13)
Asian	228 (24)	116 (12)	397 (42)	80 (8)	121 (14)
Black	65 (17)	39 (10)	172 (44)	49 (13)	64 (16)
Other	7 (16)	5 (11)	17 (38)	9 (20)	7 (16)
Language (*p* = 0.71)					
English	1024 (28)	638 (24)	1607 (60)	521 (19)	672 (25)
Non-English	199 (23)	107 (12)	309 (36)	107 (13)	127 (15)
SES quartile (< 0.001)					
High	401 (29)	227 (16)	521 (38)	129 (9)	101 (7)
Medium–high	350 (25)	209 (15)	477 (35)	159 (12)	178 (13)
Medium–low	332 (24)	170 (13)	480 (35)	171 (13)	203 (15)
Low	130 (11)	139 (12)	438 (36)	179 (15)	317 (26)
Age (*p* = 0.89)					
≤ 55	600 (23)	369 (14)	955 (36)	309 (12)	407 (15)
55 +	623 (23)	376 (14)	961 (36)	329 (12)	392 (15)
Gender (*p* = 0.12)					
Male	650 (23)	384 (14)	990 (36)	363 (13)	403 (15)
Female	573 (23)	361 (14)	926 (37)	275 (11)	396 (16)
Insurance (*p* = 0.27)					
Public	642 (22)	405 (14)	1074 (37)	368 (13)	425 (15)
Private	581 (24)	340 (14)	942 (39)	270 (11)	374 (16)

*Abbreviations*: *SES* Socioeconomic status

Table 2 presents the results of a multivariable logistic regression analysis demonstrating the association of several factors with excess delays in access after adjustment for potential confounders. The patient characteristic that was determined to be independently associated with higher odds of appointment delays was low-income status versus non-low-income status ([OR] = 3.98, 95% CI (2.01–7.92).

**Table 2:** Multi-variate analysis of potential factors for access delays^a^

Factor	Strata	% Delays	OR	95% CI	*p-*value
Race	White	16%	1.09	(0.81, 2.33)	0.35
	Latino	13%			
	Asian	14%			
	Black	16%			
	Other	16%			
Language	Non-English	15%	1.26	(0.77, 3.12)	0.74
	English	25%			
SES quartile	High	7%	3.14	(1.71, 7.50)	** < 0.001**
	Medium–high	13%			
	Medium–low	15%			
	Low	26%			
SES quartile	Non-low quartile	12%	3.98	(2.01, 7.92)	** < 0.001**
	Low quartile	26%			
Insurance	Public	15%	0.92	(0.48, 2.40)	0.51
	Private	16%			

*Abbreviations*: *SES* Socioeconomic status, *OR* Odds ratio, *CI* Confidence interval

^**a**^Defined as greater than 21 days from time of referral to consultation

## Discussion

The results of the present study demonstrate the profound impact of SES in accessing tertiary-based oncology treatment and show that the location of a patient’s residence potentially affects the expediency in which health services are obtained. These findings, obtained from a prospective registry of cancer patients referred for radiation therapy, suggest that patients from lower SES backgrounds are at higher risk for delays. More specifically, those from lower income backgrounds were more than twice as likely to be seen in excess of 21 days after referral compared to patients from higher income backgrounds. While the specific reasons underlying these disparities are speculative, the implications with respect to health equity are profound given the implications on health outcome.

Numerous studies have attempted to evaluate how income contributes to uneven access to health care [11, 12]. Given the association between income and educational status, the possibility that a lack of health awareness regarding the importance of treatment and/or the implications of a cancer diagnosis leads to delays in seeking care. As health systems are increasingly promoting digital communication and scheduling tools as a means of empowering patients, it has also been demonstrated that patients from underserved backgrounds are utilizing such platforms at disproportionately lower rates [13–15]. More recently, Alexander et al. studied the utilization of digital portals among 25,367 patients in radiation oncology and demonstrated significant disparities among patients sending portal messages to providers with lower rates identified among Black patients, those with Medicaid insurance, and females [16]. Notably, both the sending and receipt of messages from providers were significantly associated with improvements in overall survival even after adjusting for socioeconomic, disease, and treatment characteristics.

Our findings also highlight the influence of social determinants in affecting access [17]. The role of logistical coordination, in particular, has been demonstrated to be vital in adhering to appointments and treatment recommendations. Potential factors that could lead to impaired access include those related to childcare, family caretaking, housing, and employment. Along these lines, the importance of geography cannot be understated as higher-quality schools and health services tend to be in more affluent neighborhoods. A person’s immediate surroundings also dictate lifestyle factors such as access to healthy foods, opportunities for physical activity, safe transportation, and other conditions such as water and air quality [18]. All of these variables can affect access to healthcare. The influence of education on health disparities related to access has also been shown [19, 20]. Patients who lack access to services focusing on awareness and prevention might not have as high of an understanding of timely care. People living in impoverished areas are also at risk for delays in care due to specific obstacles related to the inability to travel or to take time off work [21]. Indeed, patients have cited transportation and work-related concerns as a key limit on the ability to access care and treatment [22]. Recent statistics from the United States National Health Interview Survey estimate that nearly 6 million patients go without care because they cannot access transportation to their providers and that the poor disproportionately bear the burden of this problem [23].

The escalating cost of healthcare, particularly high out-of-pocket patient costs, is another well-documented access barrier, which is particularly magnified for those living in poorer areas. For example, the study of “financial toxicity” has gained increased attention given the disproportionately high amounts that many patients pay for healthcare compared to other services [24]. Data from West Health and Gallup poll found that 29% of adults reported putting off medical treatment because of out-of-pocket costs between 2001 and 2021 [25]. While 34% of Americans with an annual household income of less than $40,000 were likely to skip or delay healthcare for a serious medical condition, a considerable proportion of higher earners (18%), defined as those with household income greater than $100,000, also stated the same. According to 2023 data from the Commonwealth Fund, the United States has the starkest income-based health disparities compared to other similarly developed nations [26]. Indeed, 46% and 27% of American adults skipped have skipped a medical visit, test, treatment, follow-up, or prescription fill within the last year solely because of cost among low-income and high-income earners, respectively. Financial toxicity is especially relevant in radiation oncology due to rising costs associated with expensive technology and the time required for treatment. Studies have also shown that with a diagnosis of cancer, the risk of bankruptcy and/or home foreclosure increases significantly [27].

Psychosocial factors can also contribute to access delays. Medical appointments can routinely conjure up emotions of fear and despair that can be exacerbated in certain underserved communities. Studies have shown that the observed delays with some medical services might be due to anxiety and perceived discomfort. A lack of trust, which can compound the fear of having to navigate a complex system has also been shown to be prevalent among the underrepresented including those of lower SES classes [28]. Although non-English speaking patients would be assumed to be at higher risk in facing access barriers, it is also plausible that resources, financial means, and/or family can circumvent these obstacles in the real world.

On the most basic level, when patients delay cancer treatment, continuity of care is interrupted; as a result, cancers might not be effectively monitored nor treated, and the risk of complications from neglect increases. Additionally, the phenomenon of up-staging (i.e. progression of disease to more advanced stages) has been well-described and is one reason why underrepresented minorities may have higher mortality rates from cancer [29]. Studies have shown that even short delays in initiating treatment for cancer adversely affects survival [30]. From a radiobiological standpoint, tumor cells have the potential to grow into more hypoxic and resistant phenotypes with delay [31].

The use of zip code data as a surrogate for SES in this study could introduce inaccuracies, and lead to misrepresentations of a patient’s actual income status and/or educational level. The latter is especially important as studies have consistently shown that access improves as one’s education rises. Some have suggested that the Area Deprivation Index (ADI), which utilizes American Community Service data, could be a more suitable measure of SES [32]. However, the ADI incorporates data from set 5-year periods, which could lead to drawing conclusions based on outdated information. This is especially pertinent given trends towards migration during the COVID-19 pandemic [33].

Another limitation of this study is that the exact reasons for access delays could not be determined with certainty. Further, it was not possible to show how these access disparities affected such endpoints related to cancer outcomes. Others, however, have documented the link between access and survival [34]. Lastly, the decision not to analyze time to initiation of radiation treatment as an endpoint was made due to several considerations. First, not all patients seen for consultation proceeded with treatment; second, since treatment was often multi-disciplinary in nature, confounding factors related to the sequencing of other modalities such as surgery and/or chemotherapy would be introduced; third, the acuity and urgency of the diseases observed were highly variable which might have influenced decisions regarding the timing of treatment initiation.

Lastly, it must be acknowledged that the current study population originated from a single-institutional setting and only represented 58% of all total patients referred. The reasons why patients were not scheduled or failed to appear for appointments are speculative but undoubtedly multifactorial. While many of these patients sought second- and third-opinion consultations at other tertiary-based centers in the metropolitan region and initiated treatment elsewhere, others may have experienced barriers in proceeding with care due to logistical factors. These considerations are noteworthy because studies have shown that higher quality hospitals tend to be located in more affluent neighborhoods [35, 36]. As such, it is possible that non-patient factors related to administrative burden, provider capacity, and resources could have impacted these findings [37].

The results of the present series are particularly instructive because they illustrate how SES and the associated social determinants of health potentially impact access to radiation oncology services. However, it is acknowledged that the decision to use SES (based on geographical zip code) as the primary basis for analysis is imperfect. While others have attempted to correlate insurance status with care delays, inconsistencies exist in the literature on just how impactful that variable might be in predicting access [38, 39].

Notably, significant barriers were identified for patients of lower SES background in accessing appointments in an expedient manner for higher-level oncology care. The results demonstrating that certain segments of the population are at risk for access impairment, as measured by delays in appointment scheduling, are consistent with previous findings showing that the same groups are at risk for missed appointments (“no shows”) [40]. Along these lines, access in healthcare can be evaluated using a variety of indicators, including utilization rates, outcome indicators, and patient-reported experiences. While it must be noted that these all measure whether individuals can obtain needed care in a timely manner and achieve positive health outcomes, it is uncertain which might be the optimal metric to track for performance improvement. While the reasons for access delays remain speculative, efforts to ensure that care is equitable and culturally competent must be improved so that the playing field for disadvantaged communities is levelled. This will require engagement from all stakeholders and the appropriate resource allocation to address issues related to access disparities. The promotion of community-based, culturally tailored educational programs including the development of financial navigation and patient-assistance programs will be important to address the challenges facing vulnerable populations.

## Conclusions

The influence of SES in contributing to access delays was demonstrated among cancer patients. Specifically, this study showed that the geographical zip code in which patients strongly influences their ability to access care and is associated with delays. Further research is needed to better understand the underlying causes of the disparities that were identified. Future interventions aimed at promoting equity by developing resources for at risk-populations are urgently warranted.

## Data Availability

Data will be available upon request to the corresponding author.

## Abbreviation

SES: Socioeconomic status
